# Salivary cortisol as an indicator of adrenocortical function in healthy infants, using massage therapy

**DOI:** 10.1590/S1516-31802005000500003

**Published:** 2005-09-01

**Authors:** Monalisa de Cássia Fogaça, Werther Brunow Carvalho, Clóvis de Araújo Peres, Mayra Ivanoff Lora, Lilian Fukusima Hayashi, Ieda Therezinha do Nascimento Verreschi

**Keywords:** Pituitary-adrenal system, Massage, Infant, Circadian rhythm, Stress, Sistema Hipófise-Adrenal, Massagem, Lactente, Ritmo circadiano, Estresse

## Abstract

**CONTEXT AND OBJECTIVE:**

The evaluation of adrenocortical function with the use of therapeutic massage has been little studied in Brazil. The purpose of this study was to evaluate the salivary cortisol levels before and after Shantala massage therapy on healthy infants.

**DESIGN AND SETTING:**

Prospective case series, in a public nursery, in São Paulo.

**METHODS:**

Saliva was obtained from 11 infants at the times of 8:00-9:00 a.m. and 4:00-5:00 p.m. in a nursery and 9:00-10:00 p.m. at home. They received a 15-minute therapeutic massage on two consecutive days, and saliva was collected before and after the massage. The procedure was repeated after a one-week interval. Cortisol values (intra-assay < 5%; inter-assay < 10%) at different times of the day were compared by ANOVA.

**RESULTS:**

The mean cortisol values (nmol/l ± SD) on the first day were: morning (M) = 14.1 ± 5.7, afternoon (A) = 8.3 ± 2.7, night (N) = 3.3 ± 1.1; after two consecutive days of therapeutic massage: M = 22.3 ± 13.5, A = 13.4 ± 6.0, N = 5.8 ± 3.5; after a one-week interval: M = 15.8 ± 7.7, A = 14.3 ± 7.7, N = 3.4 ± 2.0.

**CONCLUSION:**

There was a modification in the salivary cortisol values following massage, thus reflecting possible adaptation of the hypothalamic-pituitary-adrenal axis.

## RESUMO

**CONTEXTO E OBJETIVO:**

A avaliação da função adrenocortical com uso da massagem terapêutica no Brasil tem sido pouco estudada. O objetivo foi avaliar os níveis de cortisol salivar antes e após a massagem terapêutica Shantala em lactentes sadios.

**TIPO DO ESTUDO E LOCAL:**

Prospectivo/série de casos, numa creche pública em São Paulo.

**MÉTODOS:**

Amostras de saliva foram obtidas de 11 lactentes durante um dia entre 08h00 e 09h00, 16h00 e 17h00 na creche, e entre 21h00 e 22h00 na residência. Durante dois dias consecutivos os lactentes receberam 15 minutos de massagem terapêutica, coletando-se a saliva antes e após a massagem. O procedimento foi repetido após intervalo de uma semana. Os valores de cortisol (duplicatas; coeficiente de variação intra e interensaio < 5% e < 10%) nos diferentes períodos do dia foram comparados por análise de variância com medidas repetidas (ANOVA) e análise descritiva.

**RESULTADOS:**

Os valores médios no primeiro dia (± desvio padrão) de cortisol (nmol/l) foram: manhã (M) = 14,1 ± 5,7; tarde (T) = 8,3 ± 2,7; noite (N) = 3,3 ± 1,1; e, após dois dias consecutivos com massagem terapêutica, foram: M = 22,3 ± 13,5; T = 13,4 ± 6,0; N = 5,8 ± 3,5, respectivamente. Após intervalo de uma semana, foram: M = 15,8 ± 7,7; T = 14,3 ± 7,7 e N = 3,4 ± 2,0 nmol/l.

**CONCLUSÃO:**

Houve modificação nos valores de cortisol salivar pós-massagem, refletindo possível adaptação do eixo hipotalâmico-hipofisário-adrenal nos lactentes.

## INTRODUCTION

The practice of massage is a very ancient activity that spread out from the Orient.^1^ Over recent years, it has been presented in the West as an alternative therapeutic method that gives rise to certain proposed effects. Among the uses that have been put forward, there has been increasing development of techniques for its use on babies. One of the most widely-used of these massage techniques in the hospital environment in Brazil is Shantala,^2^ which was brought from India by the French doctor Frédérick Leboyer.^1^ Shantala massage is one of the oldest and most traditional therapies, especially in the Kerala region of southern India, where it first became widely used among the population. Initially, it was used by monks in monasteries, and subsequently it grew into a tradition that was transmitted naturally and progressively from mother to daughters, when they first became pregnant. In the 1960s, while visiting India, Leboyer observed a young woman serenely concentrating on massaging her baby while sitting on the floor with the child over her legs.^1^

According to Mathai, the use of massage therapy on preterm and full-term babies was associated with weight gain and superior behavioral maturation using Brazelton's scale.^3,4^ Although the effects of massage therapy on psychological maturation are not well known, the autonomous nervous system and the hypothalamic-pituitary-adrenocortical axis are the mediators of its effects.^4–6^

Assessing stress hormone levels (salivary cortisol) and/or neuropeptide pain levels (substance P) during stressful medical procedures could provide additional measurement of the effects of massage therapy as an adjunct to the standard management of seriously ill patients.^7^

## OBJECTIVE

With the possibility in mind that tactile-kinesthetic stimulation could be used to diminish distress among hospitalized infants (e.g. in pediatric intensive care units), we evaluated the levels of salivary cortisol before and after Shantala massage therapy on healthy infants. Through this, we aimed to obtain information that could contribute towards understanding the role of the hypothalamic-pituitary-adrenal axis in relation to traumatic stress.

## PATIENTS AND METHODS

The effects of Shantala massage therapy on salivary cortisol levels were ascertained for 11 infants (4 females and 7 males), aged 4 to 6 months (median age: 5 months) who were regularly enrolled in a public nursery, in São Paulo, Brazil. The Ethics Committee of the university approved the protocol and the parents gave written consent for their child's participation (Protocol no. 033-01; Research Ethics Committee of Universidade Federal de São Paulo — Escola Paulista de Medicina).

Saliva samples were obtained at three times during a single day: between 8:00 and 9:00 a.m. and between 4:00 and 5:00 p.m. at the nursery, and between 9:00 and 10:00 p.m. at home. This was done on the day before the first massage session and after it. Saliva was collected (one hour prior to the massage), with due regard for the meal times established at the daycare nursery (morning and afternoon) and the night bottle. The infant's oral cavity was previously cleaned with gauze, to avoid any remains of milk.^8^ With a swab moistened in lemon juice, saliva was stimulated and a sample of 500 μl was aspirated into an insulin syringe, and stored at −20° C until the radioimmunoassaying of cortisol.

The massage procedures and saliva sample collections were performed between June 2001 and April 2002. During the autumn and winter, a heater was used to maintain room temperature at approximately 25° C during the massage.

The infants received two standard 15-minute massages applied by the author (MCF) on two consecutive days, and also after a one-week interval, in the morning and in the afternoon. During the day, two massage sessions were performed, one in the morning after the saliva had been collected and the other in the afternoon. At night there was no massage, only saliva collection. The procedure utilized is described in the following.^1^

**Figure 1:**
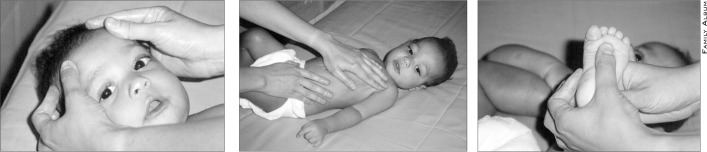
Pictures from moments during Shantala massage therapy on an infant (published with permission from the mother).

For the face, use your fingertips, starting in the middle of the forehead, moving towards the sides (temples); then, in circular movements, slide from the temples to the mandible region; and again using the fingertips, slide towards the nose, cheek, jaw-bone and chin. For the legs, hold the child's leg with your hands and make synchronized movements from the hip to the foot, pressing lightly and using “spiral” movements, sliding from the hip to the foot; then, using your thumbs, massage each toe. For the arms, start by sliding your hands from the shoulders to the fingertips, as done for the legs. For the back and shoulders, with your palms open, slide along the whole back, from the neck to the hip; alternate your hands going backwards and forwards, sliding from the neck to the hip and vice-versa; your hands come and go, moving up and down (Figure 1).^1^

During and after the massage, the children were observed by the same author (MCF), regarding their alertness/drowsiness, movement (agitation/relaxation), babble/cry and distress parameters.^4^

The assay was performed in duplicate, on two samples of 100 μl of saliva without any previous extraction. The antiserum used was a rabbit antiserum for cortisol-3-carboxymethyloxime-bovine serum albumin conjugate, which is highly specific for cortisol (100%), with low cross-reactivity for cortisone (15%), corticosterone (3%) and 11-deoxycortisol (2%), but without significant cross-reactivity (< 1%) for other steroids. The mean intra-assay and inter-assay coefficients of variation were < 5% and < 10%, respectively.^9,10^

The salivary cortisol values from the different times of the day were compared by analysis of variance with repeated measurements (ANOVA) and descriptive analysis: median, mean and standard deviation, to evaluate the differences between the mean salivary cortisol values obtained in the morning, afternoon and night. The significance level adopted was p < 0.10.

## RESULTS

**Figure 2:**
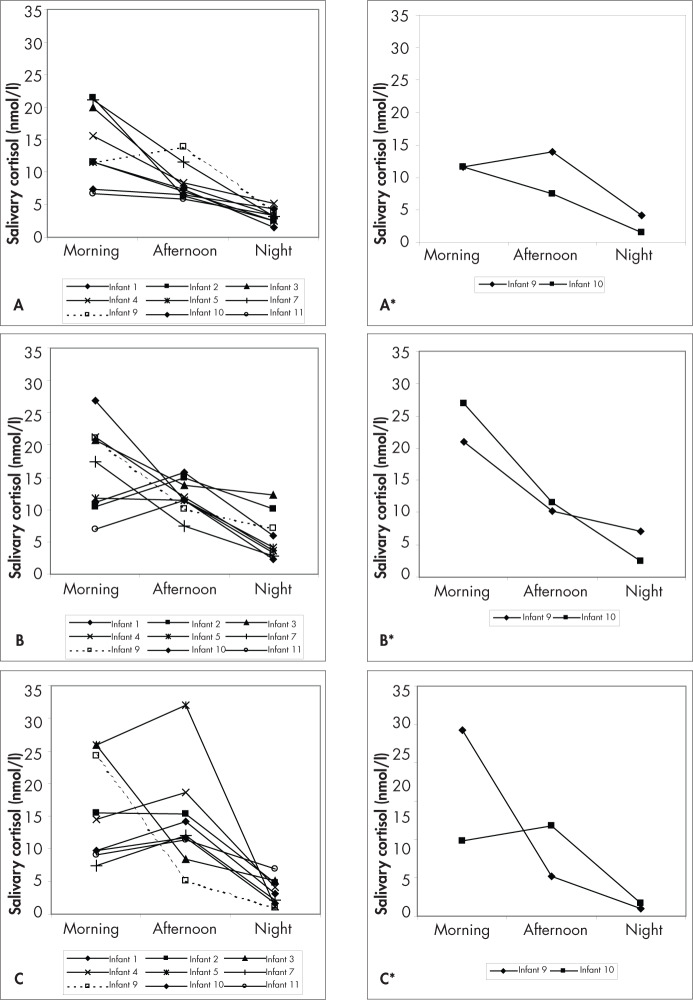
Circadian rhythm variation in salivary cortisol among healthy infants (#6 and #8 excluded) over the day before massage therapy (A); over the day after two consecutive days of 15 minutes of standard massage therapy (B); over one day after a one-week interval (C); and individual data for the twin brothers #9 and #10 (A*, B*, C*). Graphs A*, B* and C* show the individual variability in these twins, regarding the timing of the appearance of cortisol circadian rhythm and responsiveness of the hypothalamic-pituitary-adrenal axis. The massage therapy gave different results for each of the twin brothers. This example of twin brothers perhaps demonstrates that massage therapy produces different adaptive effects in the hypothalamic-pituitary-adrenal axis.

Initially, the data underwent descriptive analysis, consisting of graphs and statistics (mean and standard deviation). The individual values for the variation in the salivary cortisol levels (nmol/l) of nine infants are shown in Figure 2. The data on the other two infants (#6, undergoing corticotherapy, and #8, subsequently diagnosed with Dubowitz syndrome) were not utilized.

On the day before performing the first massage, the mean values (± standard deviation) were: morning (M) = 14.1 ± 5.7 nmol/l; afternoon (A) = 8.3 ± 2.7 nmol/l; night (N) = 3.3 ± 1.1 nmol/l. After two consecutive days of massage, the mean values were: M = 22.3 ± 13.5 nmol/l; A = 13.4 ± 6.0 nmol/l; N = 5.8 ± 3.5 nmol/l, respectively. After an interval of one week, the procedure of obtaining the samples and massage was repeated, and the results were: M = 15.8 ± 7.7 nmol/l; A = 14.3 ± 7.7 nmol/l; N = 3.4 ± 2.0 nmol/l (Table 1). After two consecutive days of massage therapy, all nine of the infants whose data presented altered levels of salivary cortisol, but a statistically significant difference (p ≤ 0.060) was observed only in the afternoon period.

**Table 1.** Mean salivary cortisol concentration (nmol/l) in nine healthy infants before and after two consecutive days of massage therapy and after another massage therapy session applied one week later

Period	Salivary cortisol concentrations (standard deviation)		
	Before massage therapy	After two days of massage therapy	After further massage one week later
Morning	14.1 (5.7)	22.3 (13.5)	15.8 (7.7)
Afternoon	8.3 (2.7)	13.4 (6.0)	14.3 (7.7)
Night	3.3 (1.1)	5.8 (3.5)	3.4 (2.0)

Comparison of the basal mean cortisol value for the morning period (14.1 nmol/l) with the post-massage mean values, obtained on two consecutive days of massage (22.3 nmol/l) for the same time of the day showed statistical similarity between the values (f = 2.126; p ≤ 0.147).

A statistically significant difference (f = 3.602; p ≤ 0.060) was observed between the mean basal value for the afternoon salivary cortisol (8.3 nmol/l) and the mean value obtained for the same time of the day, after two consecutive days of massage (13.4 nmol/l). No statistically significant difference (f = 2.405; p ≤ 0.123) was found between the mean basal value for the night period (3.3 nmol/l) and the post-massage mean value (5.8 nmol/l) for the same time of the day, after two consecutive days with massage therapy. The saliva collection was performed before and after each massage session.

The timing of the appearance of cortisol circadian rhythm and responsiveness of the hypothalamic-pituitary-adrenal axis presented individual variability, which was demonstrated in this study in infants #9 and #10, who were twin brothers.

With regard to the infants’ behavioral state, during and after the massage therapy, no sign of distress was observed. On the contrary, seven infants (64%) started to fall asleep, and the others remained calm and in a state of alertness.

After a one-week interval, although the circadian rhythm was not lost, the post-massage cortisol values became modified for all infants.

## DISCUSSION

The appearance of cortisol circadian rhythmicity is usually observed in the first stages of the child's development.^11–13^ Depending on the child being evaluated, this rhythmicity can appear between the 8^th^ and 20^th^ week of life,^10,14^ and this was also found in the present study.

Although most of the infants in our study presented an established cortisol circadian rhythm, two presented the beginning of this synchronization and two other infants did not present circadian rhythmicity.

After the massage therapy, higher (but not statistically significant) levels of cortisol were observed in the morning in most infants, and in the afternoon in two of them, after two consecutive days of massage. This change apparently began to take place at night, with incomplete suppression of the axis at this time, thereby leading to raised cortisol levels in the mornings of the days with massage. Repeating the procedure, after a one-week interval, higher cortisol levels were observed in the morning and in the afternoon.

Kuhn et al.^6^ also reported that, following tactile-kinesthetic stimulation, the levels of urinary cortisol were high in pre-term babies. This demonstrated the relationship between the sympathetic nervous system and the effects of tactile-kinesthetic stimulation. Gunnar^5^ and Gunnar et al.^4^ reported that there was an association between adrenocortical activity and behavioral responses. The present study confirms both observations, since Shantala massage therapy altered the cortisol secretion levels, thereby probably altering the reactivity of the hypothalamic-pituitary-adrenal axis.

From the repeated measurement variance analysis (ANOVA), a statistically significant difference was observed between the mean basal value for the afternoon period and the post-massage value for the same time of the day. This seems to indicate that the massage effects performed in the afternoon could also have been influenced by the variables of feeding times (lunch and/or bottle) and afternoon nap. According to previous studies,^13,15–17^ feeding, as well as sleep, can alter the cortisol levels.

Although the children studied were part of a group of healthy infants, this research aimed to obtain information on the effects of massage therapy, so as to understand the role of the hypothalamic-pituitary-adrenal axis in the event that there is any alteration that causes traumatic stress.^7^

The present data suggest that the circadian maturation of the hypothalamic-pituitary-adrenal axis occurred early, during the first months of life, thereby organizing and revealing the cortisol rhythmicity.

Even considering the small size of the sample studied, it could be seen that Shantala massage therapy did not cause any damage to the hypothalamic-pituitary-adrenal axis. However, the present data are conditioned by the genetic diversity and/or environmental influences on Brazilian infants, in relation to other population groups. Therefore, for correct interpretation of the results, the individual variations in cortisol secretion relating to Shantala massage therapy need to be considered. The effect on hospitalized children (for example, in pediatric intensive care units) also needs to be considered, since such children may be in situations that are stressful because they are deprived of their mother's presence.

## CONCLUSION

There was a modification in the salivary cortisol values following massage, thus reflecting possible adaptation of the hypothalamic-pituitary-adrenal axis.
